# Improved estimation of aboveground biomass of regional coniferous forests integrating UAV-LiDAR strip data, Sentinel-1 and Sentinel-2 imageries

**DOI:** 10.1186/s13007-023-01043-9

**Published:** 2023-06-30

**Authors:** Yueting Wang, Xiang Jia, Guoqi Chai, Lingting Lei, Xiaoli Zhang

**Affiliations:** 1 Beijing Key Laboratory of Precision Forestry, College of Forestry, Beijing Forestry University, Beijing, 100083 China; 2 Key Laboratory of Forest Cultivation and Protection, Ministry of Education, Beijing Forestry University, Beijing, 100083 China; 3 National Engineering Laboratory for Satellite Remote Sensing Applications, Aerospace Information Research Institute, Chinese Academy of Sciences, Beijing, 100094 China

**Keywords:** UAV-LiDAR strip data, Point-line-polygon framework, LiDAR sampling plots, Multi-scale wavelet transform, Aboveground biomass

## Abstract

**Background:**

Forest aboveground biomass (AGB) is not only the basis for estimating forest carbon storage, but also an important parameter for evaluating forest carbon cycle contribution and forest ecological function. Data saturation and fewer field plots limit the accuracy of AGB estimation. In response to these questions, we constructed a point-line-polygon framework for regional coniferous forests AGB mapping using field survey data, UAV-LiDAR strip data, Sentinel-1 and Sentinel-2 imageries in this study. Under this framework, we explored the feasibility of acquiring the LiDAR sampling plots using the LiDAR sampling strategy consistent with the field survey, and analyzed the potentials of multi-scale wavelet transform (WT) textures and tree species stratification for improving AGB estimation accuracy of coniferous forests in North China.

**Results:**

The results showed that UAV-LiDAR strip data of high density point clouds could be used as a sampling tool to achieve sample amplification. Experimental comparison results showed that the Sentinel-based AGB estimation models incorporating the multi-scale WT textures and SAR data performed better, and the model based on coniferous forests tree species significantly improved the performance of AGB estimation. Additionally, the accuracy comparison using different validation sets indicated that the proposed LiDAR sampling strategy under the point-line-polygon framework was suitable for estimating coniferous forests AGB on a large area. The highest accuracy of AGB estimation of larch, Chinese pine and all coniferous forests was 74.55%, 78.96%, and 73.42%, respectively.

**Conclusions:**

The proposed approach can successfully alleviate the data signal saturation issue and accurately produce a large-scale wall-to-wall high-resolution AGB map by integrating optical and SAR data with a relative small number of field plots.

## Highlights

1. The LiDAR sampling strategy consistent with the field survey under the point-line-polygon framework for wall-to-wall large-scale coniferous forests AGB mapping combining limited field plots, UAV-LiDAR strip data, and multi-sensor satellite imagery is proposed.
2. Build and compare different AGB estimation models of coniferous forests under stratification of tree species and non-stratification.
3. Analyze the improvement of AGB estimation accuracy and the reduction of the data saturation issue by incorporating the multi-scale wavelet transform features of Sentinel-1 and Sentinel-2 data.

## Background

Forest ecosystem, as an indispensable part of terrestrial ecosystem, has strong carbon sink capacity and plays an irreplaceable role in carbon cycling [29, 38, 51]. As the basis of estimating forest carbon storage, forest biomass has been listed as a necessary parameter for monitoring forest carbon sink capacity and assessing carbon budget [20, 40]. According to the 9th Chinese Continuous Inventory of National Forest Resources Statistics [1], the total forest area of China is about 2.204462 × 10^8^ ha, accounting for 22.96% of the country’s total area. Inner Mongolia Autonomous Region, as one of the important forest zone in China, has about 2.61485 × 10^7^ ha of forest, ranking first in China. And the coniferous forests cover an area of about 5.6533 × 10^6^ ha, accounting for 32.62% of Inner Mongolia’s total forest area. Therefore, it is of great significance to accurately estimate the biomass of coniferous forests in this region for assessing carbon storage in China.

Traditionally, forest aboveground biomass (AGB) is estimated with allometric growth equation by measuring tree height and diameter at breast height in field survey, which could provide the accurate result at plot scale [28, 52]. This approach is time-consuming and laborious and impossible to map AGB distribution accurately, especially on a large area [37]. Remote sensing technique, as an objective, continuous and repeatable observation method, has been widely used in forest AGB estimation [32, 36, 39, 50, 55]. The common methods focus on statistical models, that is, the estimation model is established combining field-measured plots and variables derived from remote sensing data, and then extrapolated to the whole study area [31]. Under this method, the number and distribution of the field plots have a great impact on the AGB estimation accuracy. Although multi-spectral sensors have been widely used in forest AGB estimation [3, 11, 14, 39, 41], spectral saturation limits estimation accuracy [53, 54]. As one type of active remote sensing sensors, Synthetic aperture radar (SAR) has certain penetrability to forest canopy, and is sensitive to water content in vegetation and not affected by clouds [17, 35]. Although SAR can improve the saturation value of AGB, it still cannot completely solve this problem [24, 34]. Light Detection and Ranging (LiDAR), as another active sensor, is not limited by data saturation and can accurately describe the three-dimensional structure information of forests [12, 23]. It is the most effective and accurate remote sensing technology for estimating forest AGB at present [16]. However, due to its high cost of data acquisition, airborne LiDAR data are limited to map forest AGB in a large area [42].

Progress has been made by integrating multi-sensor remote sensing data for producing a wall-to-wall forest AGB map on a large-scale [6, 10, 26, 33, 45, 49]. An approach called a point-line-polygon framework, in which LiDAR data serves as intermediate data to link field plots with satellite imagery, has been applied to forest biophysical attributes estimation at a large-scale, such as forest volume stock, tree height and forest biomass [9, 48]. The point-line-polygon framework is mainly divided into two stages. The first stage is to establish the LiDAR-based model to relate field plots and LiDAR metrics, and estimate forest biophysical attributes throughout the LiDAR coverage. Second stage extrapolated forest biophysical attributes to the broader coverage using the equation between the LiDAR derived attributes and satellite imagery. LiDAR data are used as intermediate samples to extrapolate AGB estimation from plot-level to a wall-to-wall coverage. Under the point-line-polygon framework, the advantages of the integration of LiDAR data and multi-sensor imagery can be synergistically utilized, and LiDAR can be used as a sampling tool to alleviate the problem of limited field plots through appropriate sampling. The point-line-polygon framework has yielded accurate results for forest parameters on a large-scale. However, there are rarely researches on the construction of the point-line-polygon framework by integration of airborne LiDAR strip data and multi-source imagery, especially for coniferous forests AGB estimation.

Features extracted from multi-sensor data are the foundation of AGB modelling. In addition to features reflecting spectral information such as spectral reflectance and vegetation index, remote sensing images contain abundant texture information, including spatial domain texture and frequency domain texture [8, 15, 30, 31]. Many studies have shown that spatial domain textures extracted from both optical and SAR data could help improve the estimation accuracy of forest AGB [22, 31, 43, 44]. The spatial domain texture is represented by the grayscale distribution of pixels and their surrounding spatial neighborhoods, while the frequency domain texture is to transform the image into frequency domain and derive its texture from the spectrum. The frequency domain texture can be obtained by two-dimensional Fourier transform or two-dimensional wavelet transform (2-D WT), which have been applied in classification and estimation of forest parameters with good results [4, 5, 18, 47]. However, the potential of frequency domain features for forest AGB estimation is rarely discussed.

Here, we construct a point-line-polygon framework for large-scale coniferous forests AGB mapping combining limited field plots, Unmanned Aerial Vehicle LiDAR (UAV-LiDAR) strip data, optical and SAR imagery. The specific objectives of the study are as follows: (a) to evaluate the feasibility of estimating coniferous forests AGB using the proposed LiDAR sampling strategy under the point-line-polygon framework; (b) to build and compare the AGB estimation models of coniferous forests tree species and all coniferous forests; (c) to analyze the effect of AGB estimation accuracy by incorporating the multi-scale WT textures.

## Results

### LiDAR strip coverage AGB estimation

The LiDAR-based AGB models of the first stage were established by using field plots and LiDAR metrics, and assessed by the leave-one-out cross-validation method. Table 1 summarizes the results of the optimal combination of variables, modelling and validation for AGB estimation models of the larch, Chinese pine and non-stratification. For both stratification and non-stratification situations, the accuracies of these LiDAR-based AGB estimation models were high. The determination coefficients R^2^ of the LiDAR-based models were all higher than 0.8, and the correlation coefficients r between the estimated AGB_LiDAR_ and the field AGB were also all greater than 0.8. All the LiDAR-based AGB models based on tree species perform better than the one under non-stratification, indicating that stratification of tree species was effective in improving the accuracy of AGB estimation. Among the models based on tree species, the accuracy of the larch AGB model with only two variables D09, Hmean (R^2^ = 0.923, RMSE = 13.92 Mg/ha, MAE = 10.88 Mg/ha, rRMSE = 12.21%, r = 0.953) is the highest.

**Table 1:** The performance measures of LiDAR-based AGB models built by UAV-LiDAR metrics

Category	Selected variables	R square	RMSE (Mg/ha)	rRMSE (%)	MAE (Mg/ha)	r
Larch	D09, Hmean	0.923	13.92	12.21	10.88	0.953
Chinese pine	D10, Hmean, G.F, I.ske	0.859	19.11	12.49	14.04	0.913
Non-stratification	Hmax, I05, H.kur, G.F, I20	0.800	24.69	18.43	19.69	0.877

**Fig. 1:**
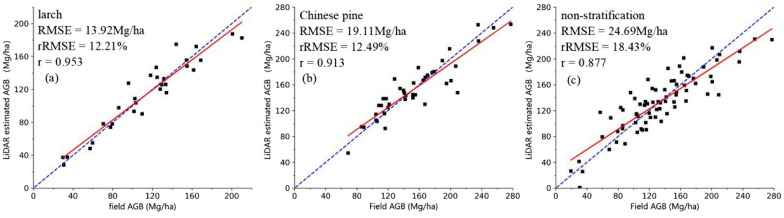
The scatterplots of field AGB and LiDAR estimated AGB

The performance of the models can be explained with the scatterplots showing the relationships between the estimated AGB_LiDAR_ and field AGB (Fig. 1). In three scenarios, there is almost no underestimation in the higher AGB range, which confirms that LiDAR could be an effective tool for plot sampling and ensures the accuracy of subsequent LiDAR sampling plots. Moreover, compared with the tree species models, the AGB model under non-stratification has a larger residual when AGB ranges are 0–70 Mg/ha and > 200 Mg/ha. These results also illustrate the importance of proper stratification based on tree species for improving AGB estimation.

### LiDAR sampling by visual interpretation

**Fig. 2:**
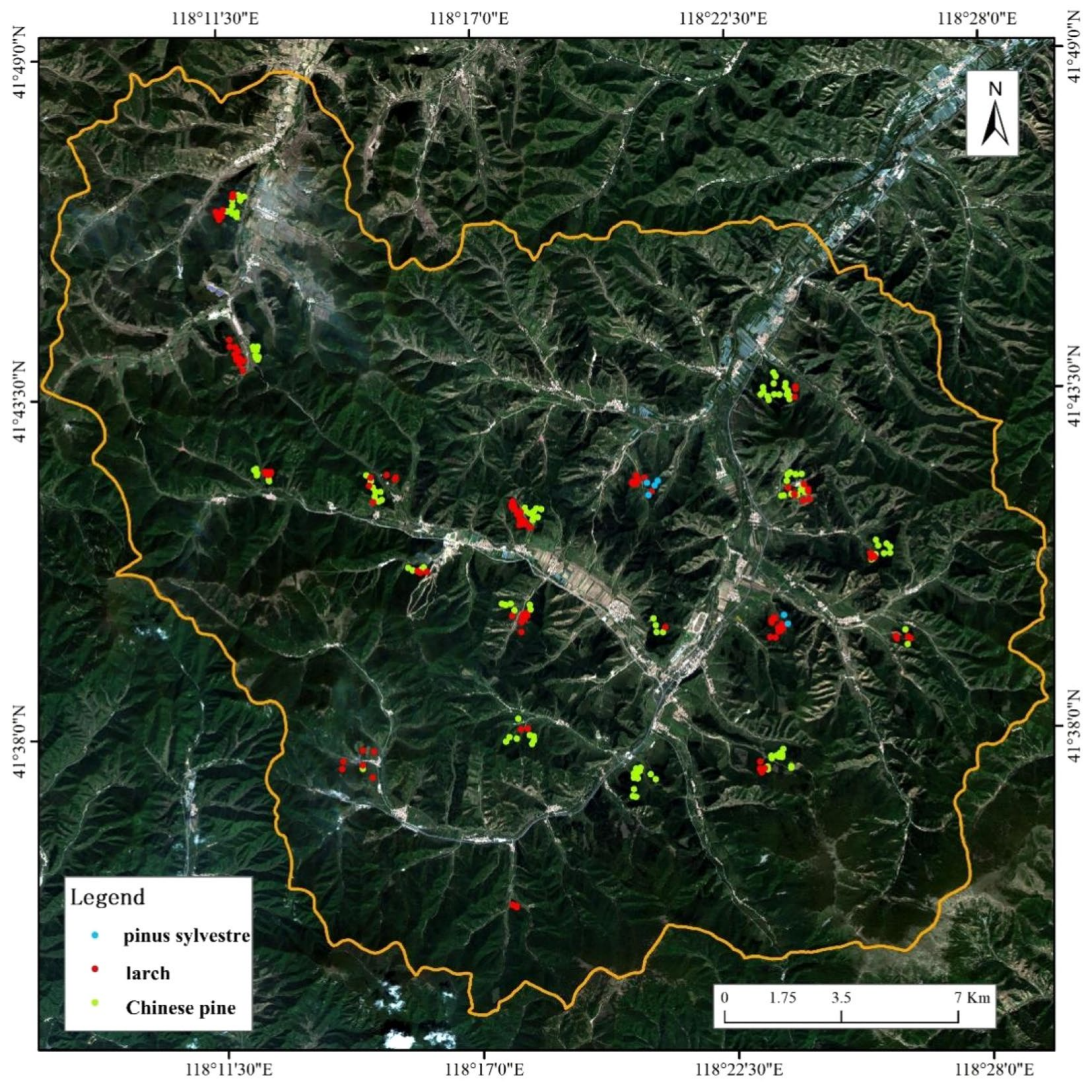
The distribution of the LiDAR sampling plots

The 25 m * 25 m LiDAR sampling plots of 115 larch, 134 Chinese pine and 6 other coniferous forests were established by visual interpretation method (Fig. 2). The size, orientation and layout principles of these plots were consistent with those of field plots. Table 2 presents a summary of the AGB_LiDAR_ of LiDAR sampling plots.

**Table 2:** Summary of the AGB_LiDAR_ of LiDAR sampling plots (Mg/ha)

Category	Number of samples	Range of AGB	Mean AGB	Standard deviation
Larch	115	11.34–192.90	83.28	44.29
Chinese pine	134	24.71–269.26	143.80	47.11
Other coniferous forests	6	10.30–145.15	85.40	54.71
Non-stratification	255	10..30–269.26	115.13	55.06

### Forest AGB modelling using Sentinel images

The results of Sentinel-based AGB models using RF algorithms in four scenarios were compared by two validation sets (Tables 3, 4). The evaluation results with LiDAR-based AGB validation set showed that the Sentinel-based AGB model all had higher overall accuracy in A, B, C and D experiments, indicating the proposed LiDAR sampling strategy under the point-line-polygon framework was suitable for coniferous forests AGB estimation.

**Table 3:** The performance measures of AGB estimation models

Experiment (category)	Selected variables	R square
A (larch)	B6, Cab, CIre, VARI, S2REP	0.598
B (larch)	Clre, Cab, a_1_B11, a_3_B4, d_1_B3	0.626
C (larch)	Clre, Cab, a_1_B11, d_1_B3, VV_mean	0.670
D (larch)	Cab, a_1_B11, d_1_B3, VV_mean, d_2 _B1_sar	0.685
A (Chinese pine)	B6, LAI, FVC, MidIR, S2REP	0.563
B (Chinese pine)	B8a, Cwc, FVC, MidIR, a_3_B8	0.573
C (Chinese pine)	B6, LAI, FVC, MidIR, VH_var	0.623
D (Chinese pine)	LAI, FVC, MidIR, VH_var, a_1 _B1_sar	0.657
A (non-stratification)	B11, Cab, CIre, Cwc, MidIR	0.516
B (non-stratification)	B12, II, FVC, Cab, h_1_B4	0.532
C (non-stratification)	Cwc, FVC, MidIR, VH, VH_var	0.603
D (non-stratification)	Cwc, FVC, MidIR, VH_var, a_1_B1_sar	0.632

**Table 4:** A summary of evaluation results of AGB models

Experiment (category)	LiDAR-based AGB				Field-based AGB			
	RMSE (Mg/ha)	MAE (Mg/ha)	rRMSE (%)	r	RMSE (Mg/ha)	MAE (Mg/ha)	rRMSE (%)	r
A (larch)	37.39	31.80	32.80	0.777	40.46	32.37	35.49	0.712
B (larch)	35.51	30.42	30.15	0.677	39.39	31.60	34.56	0.607
C (larch)	31.46	27.19	27.17	0.785	34.79	29.03	30.52	0.733
D (larch)	29.01	25.53	25.45	0.790	32.60	27.02	28.61	0.739
A (Chinese pine)	41.85	35.09	27.61	0.424	44.69	38.65	29.21	0.425
B (Chinese pine)	39.88	32.95	26.32	0.505	43.52	35.95	28.44	0.482
C (Chinese pine)	34.24	28.46	22.59	0.636	38.21	32.48	24.98	0.604
D (Chinese pine)	31.88	26.44	21.04	0.710	36.13	30.28	23.61	0.673
A (non-stratification)	44.67	35.64	33.25	0.558	47.47	37.91	35.43	0.541
B (non-stratification)	42.23	33.96	31.43	0.620	45.97	37.82	34.31	0.580
C (non-stratification)	38.83	31.77	28.90	0.682	41.09	34.02	30.67	0.675
D (non-stratification)	35.71	29.65	26.58	0.732	38.37	31.71	28.64	0.719

The highest accuracy was 74.55%, 78.96% and 73.42% for larch, Chinese pine and non-stratification using LiDAR-based AGB validation set. The evaluation accuracy of Sentinel-based AGB model using field-based AGB validation set was lower than that of LiDAR-based AGB validation set (Table 4), which was due to the accumulation of errors during the up-scaling process. However, the overall accuracy evaluated by field-based AGB validation set was also good, implying the applicability of the proposed LiDAR sampling strategy and effectiveness of the constructed point-line-polygon framework to map coniferous forests AGB over large area.

No matter stratification or not, the results illustrate that the Sentinel-based AGB models incorporated SAR data (experiment C and D) have achieved a higher accuracy (Table 4) than the models established using optical image alone (experiment A and B). The incorporation of SAR data improved the accuracy of the models by about 7% in all three scenarios, which verified the improvement of AGB estimation accuracy by fusion of optical and SAR images. Moreover, the addition of WT texture improves the accuracy of AGB estimation (B > A, D > C), especially WT feature derived from SAR.

In stratification scenario, the incorporation of WT textures from optical data has little effect on improving the performance of the Chinese pine AGB model, but better effect on improving the accuracy of the larch AGB estimation model (experiment A and B). However, the WT textures of SAR data have the opposite effect on Chinese pine and larch (experiment C and D). Among the WT textures, the approximate textures at one-level scale of optical data have the higher explanatory power for AGB estimation, and the WT textures of SAR data under VH polarization. Moreover, the Sentinel-based AGB estimation models based on tree species have better performance, which also confirms that the effectiveness of tree species stratification in improving the accuracy of AGB estimation.

**Fig. 3:**
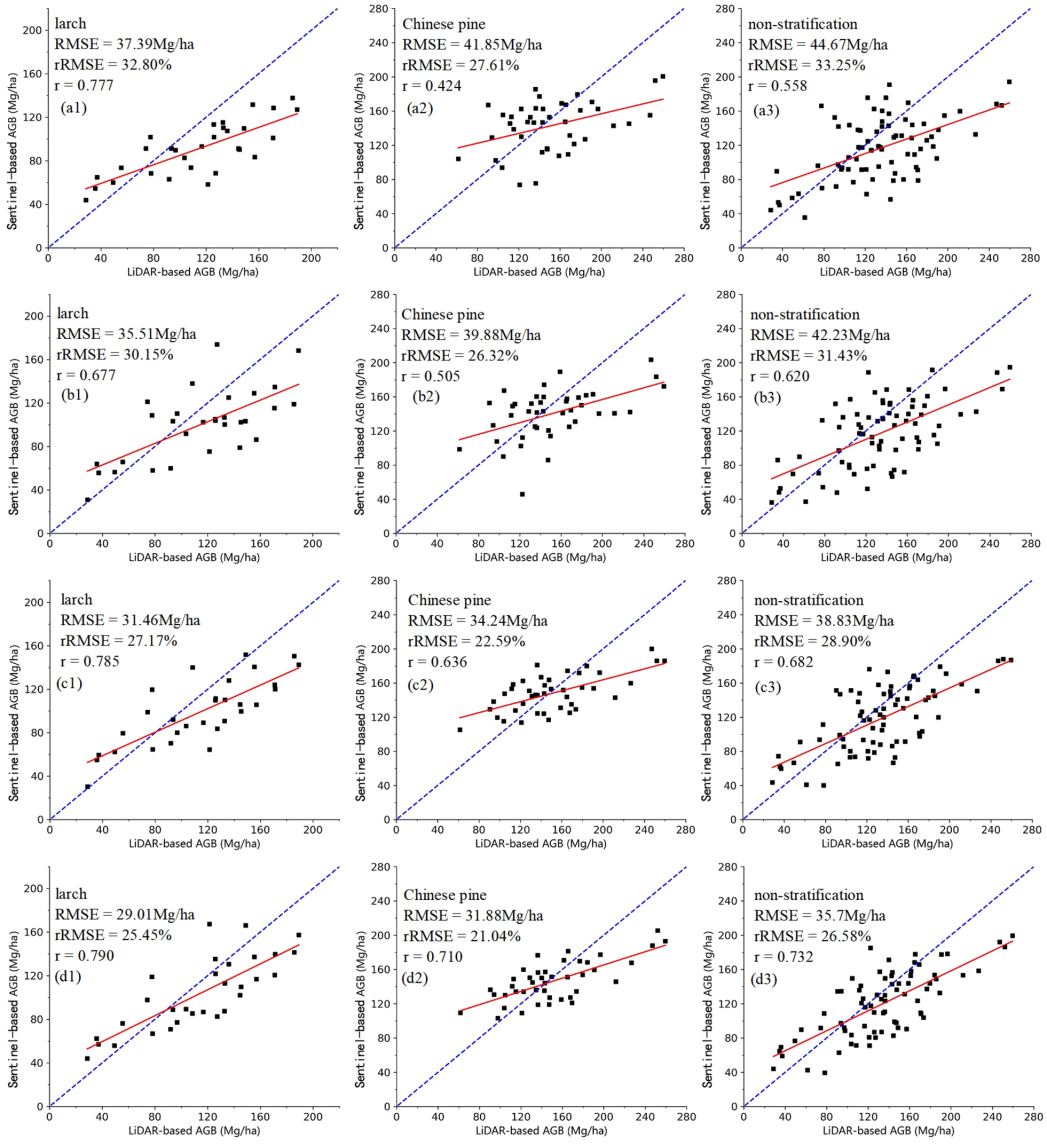
The scatterplots of LiDAR-based AGB and Sentinel-based AGB on Sentinel-based AGB models. a, b, c and d represent experiment A, B, C and D, respectively

By analyzing and comparing the scatterplots of the relationships between the estimated Sentinel-based AGB and LiDAR-based AGB (Fig. 3), it was found that in the four experiments, there are overestimation in the low AGB range (AGB < 50 Mg/ha) and underestimation in the high AGB range (AGB > 150 Mg/ha). With the addition of different types of features, the phenomenon of underestimation in the high AGB range are effectively alleviated, which means that data saturation problem could be alleviated. For larch and non-stratification scenarios, the overestimation of low AGB range hardly improved.

**Fig. 4:**
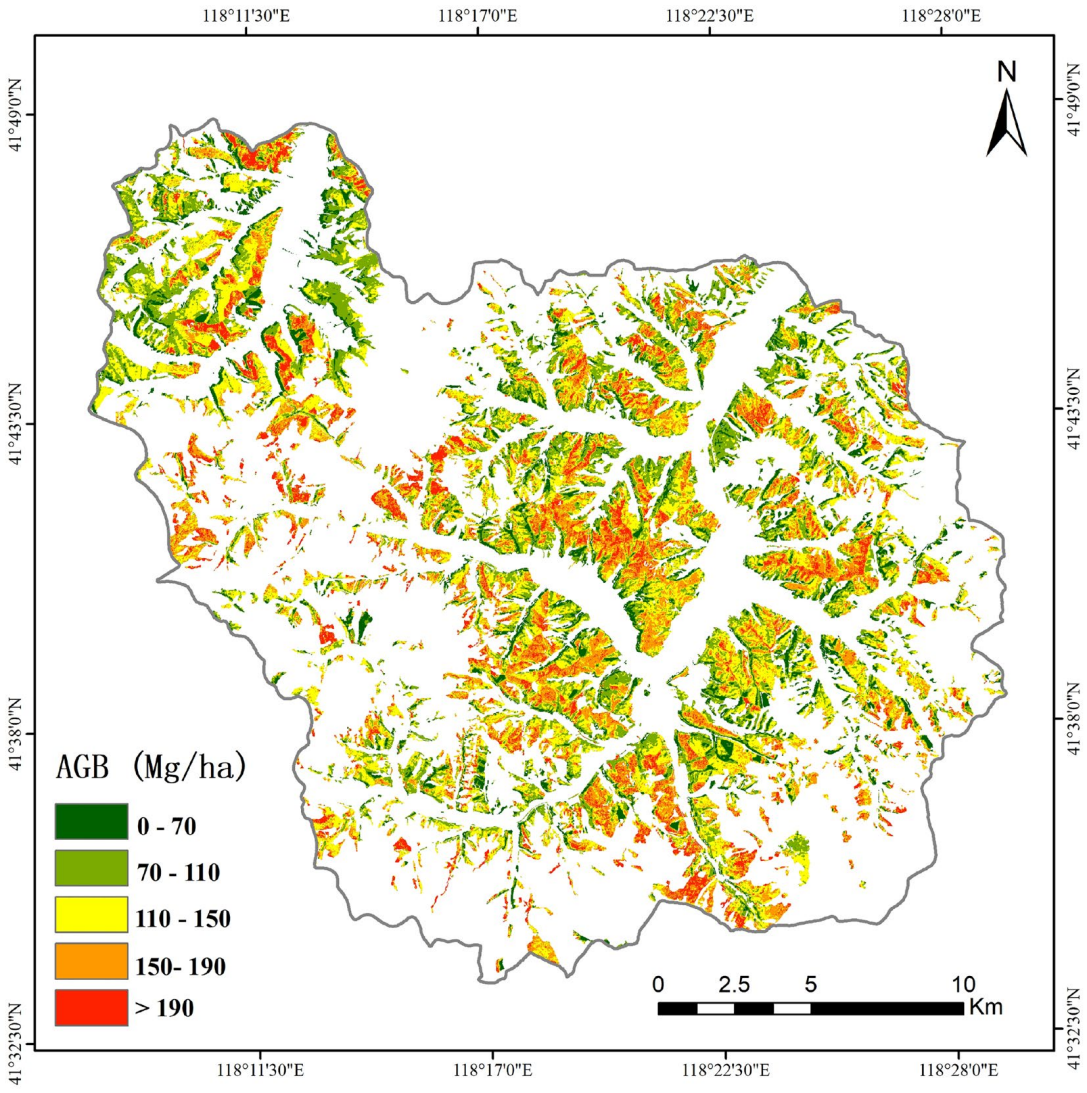
Distribution of the coniferous forests AGB in the Wangyedian Farm, North China

The coniferous forests AGB spatial distribution map was acquired using the Sentinel-based AGB estimation models in D experiment (Fig. 4). According to the attributes of tree species types from the subcompartment of Wangyedian Experimental Forest farm, the larch, Chinese pine and other coniferous forests regions were calculated by using the AGB estimation models of larch, Chinese pine and non-stratification, respectively. According to the obtained AGB distribution map, there are more pixels in the range of 110–150 Mg/ha and 150–190 Mg/ha. The distribution pattern of AGB values is similar to previous studies in [25, 50], that is, AGB values are lower in the northeastern and higher in the southern and east-central regions.

## Discussion

### The feasibility of estimating forest AGB under the point-line-polygon framework

Large-scale, high-precision AGB estimation using remote sensing method is usually limited by the difficulty in acquiring a sufficient number of evenly distributed field plots. Under the constructed point-line-polygon framework, the UAV-LiDAR strip data of high density point clouds could be used as a sampling tool for plot sampling to effectively amplify the number of field plots and reduce the workload of field measurements [33, 36]. In this study, the evaluation results of the Sentinel-based AGB models by two validation sets show that the point-line-polygon framework is suitable to estimate coniferous forests AGB over large-area without extensive field measurements. The difference in accuracy obtained using the LiDAR validation set and the field-based validation set is the error accumulation in the up-scaling process. Under different experiments, the accuracy difference is between 2 and 4.5%, which also illustrates that the error introduced by this method is controllable. Moreover, the point-line-polygon framework can better coordinate with different data sources, which makes full use of the advantages of different data sources to estimate AGB. The point-line-polygon framework provides a feasible process for large-scale and high-precision AGB estimation, so as to produce a higher spatial resolution wall-to-wall AGB map of the whole study area. In the future, when the number of acquired LiDAR sampling plots is large enough, the deep learning algorithm could be considered to build AGB estimation model and explore its potential in improving the accuracy of AGB estimation.

### UAV-LiDAR plots sampling strategy

Sampling strategies usually include systematic and stratified sampling. In systematic sampling, the sampling plots are evenly distributed, but different sampling schemes lead to large differences in results [2, 7]. In this study, the stratified sampling strategy was used to select the LiDAR sampling plots, which could meet the requirements of AGB modeling. Based on visual interpretation, LiDAR sampling plots of Chinese pine, larch and other coniferous forests consistent with field plots were selected uniformly for subsequent AGB estimation. In this way, the workload related to classification is reduced. Moreover, compared with previous researches using all pixels or the fixed-size areas under different categories to complete AGB modeling of the second stage [27, 48], our method can ensure the consistency of plots in the two stages and reduce the error during the scale conversion process in the point-line-polygon framework.

The point cloud density of UAV-LiDAR data acquired is high (> 40/m^2^), which could provide finer tree structure information, then the LiDAR-based AGB models established achieve a high accuracy. Therefore, it has great potential to effectively amplify field plots and reduce field measurement. However, the LiDAR sampling method we used may limit the number of sampling plots selected. In the following research, the point cloud density would be thinned to obtain a point cloud density threshold that can ensure high accuracy of AGB estimation. Thus, in practice, the coverage area of UAV-LiDAR data can be expanded by reducing the point cloud density while keeping the cost unchanged, so as to obtain more LiDAR sampling plots that meet the requirements.

### Potential solution to reduce the data saturation problem

Data saturation in optical data is a critical problem that restricts the improvement of AGB estimation accuracy, especially in dense forests with higher AGB [25]. This is mainly because optical data only provide spectral and horizontal spatial feature. Forest tree height, which represents the vertical structure of the forest stand, has been shown to reduce data saturation in AGB estimation [31, 46, 53]. LiDAR, Interferometric SAR and Polarimetric SAR Interferometry techniques have been utilized successfully to derive forest tree height variable. However, there are many significant limitations in extracting large-scale tree height features when applied on a large-scale, including limited data availability, high cost, technical complexity and spatially discrete data characteristics. Many other approaches, such as stratification and the use of multi-source remote sensing data [13, 21, 28], have been studied to alleviate AGB saturation.

In this study, we conducted AGB modeling experiments from the aspects of tree species stratification, collaborative optical and SAR data, and incorporating texture of the frequency domain. The results prove the positive impact of these methods on improving AGB estimation performance and alleviating data saturation. The spectral information of optical image can reflect the physiological and biochemical characteristics of forest canopy, and SAR the ability to penetrate the forest canopy, and is sensitive to water content. Generally, the fusion of optical and SAR data refers to the synergy of spectral and backscattering features. In addition to these features, multi-scale WT textures are extracted from optical and SAR data for forest AGB estimation in this study, representing texture information in the frequency domain, which is different from that in the spatial domain. The results show that WT textures can improve the saturation point, especially the WT features extracted from SAR. Therefore, the proposed methods could overcome the limitations of data acquisition and alleviate data saturation in AGB estimation, and are conducive to produce a wall-to-wall high accuracy forest AGB map on a large-scale.

## Conclusion

We have investigated the potential of the point-line-polygon upscaling framework integrating the UAV-LiDAR strip data, Sentinel-1 and Sentinel-2 imagery for estimating the coniferous forests AGB in the case study of the Wangyedian forest farm in North China. The UAV-LiDAR strip data acted as a bridge to link field plots with wall-to-wall coverage satellite imagery. And the results demonstrated that the UAV-LiDAR data of high density point cloud can be used as a sampling tool to accomplish field plots augmentation based on the high-precision LiDAR-based AGB models built by the field plots and LiDAR metrics. The integration of Sentinel-1, Sentinel-2 data and LiDAR sampling plots under the point-line-polygon framework could produce an accurate, reliable and high-resolution coniferous forests AGB map, suggesting the feasibility of this framework in estimating AGB. Furthermore, the incorporation of SAR data WT textures, and the use of stratification of coniferous forests tree species could significantly improve the AGB estimation performance. Overall, this research provides a feasible way to reduce the data saturation problem of optical data and realize an accurate AGB mapping on a large-scale by integrating different data sources. This suggests that the large-scale high-resolution mapping of forest AGB based on point-line-polygon framework by integrating UAV-LiDAR strip data and space-bone satellite images has broad application prospects, and will provide important support for carbon storage assessment and dynamitic monitoring.

## Materials and methods

### Study area

**Fig. 5:**
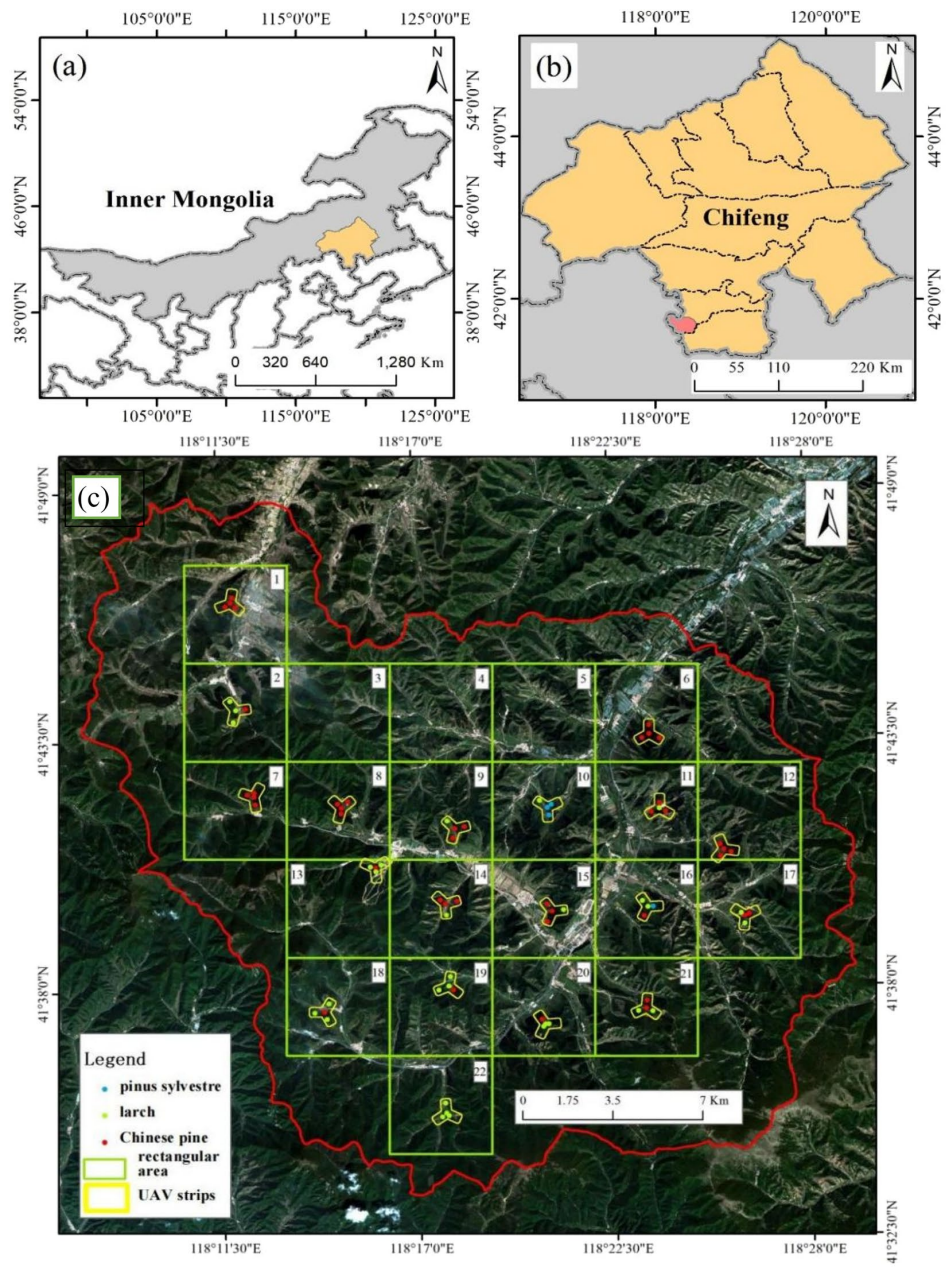
Location of the study area, the distribution of the field plots and the acquisition area of UAV-LiDAR. (**a**) and (**b**) show the locations of Chifeng City and Wangyedian Forest Farm respectively. The true color image (**c**) is composed of three bands (red, green and blue) of Sentinel-2B data

This study was conducted in Wangyedian Experimental Forest Farm (Fig. 5), with a total area of 500 km^2^, located in the southwest of Horqin Banner, Chifeng City, Inner Mongolia Autonomous Region (118° 07ʹ–118° 33ʹE, 41° 29ʹ–41° 49ʹN), China. The study area is mainly mountainous, with a north–south length of 28.1 km and an east–west length of 30.3 km. The area has average elevation of 800–1890 m, average annual temperature of 3.5–7.0 °C, and average annual precipitation of 300–500 m. The local climate is a temperate continental monsoon climate with cold and dry winters, and warm and rainy summers.

The major types of forest include coniferous plantations and secondary broadleaf forests, accounting for 55% and 40% of the total forest, respectively. The coniferous plantations mainly include larch (*Larix gmelinii (Rupr.) Kuzen.*), Chinese pine (*Pinus tabuliformis Carr.*), Scots pine (*pinus sylvestre*), and red pine (*Pinus koraiensis Sieb. et Zucc.*) And the secondary broadleaf forests mainly include birch (*Betula platyphylla Suk.*), aspen (*Populus davidiana.*), and elm (*Ulmus pumila* L.). Larch and Chinese pine account for 90% of the coniferous forests area, and are the main tree species of coniferous forests, which can represent the situation of the whole coniferous forests.

### Data

#### Field data

Field survey was carried out in the study area from mid-September to early October 2019. The systematic sampling control at forest farm level was used to arrange field plots. Firstly, Wangyedian Forest Farm were divided into 22 rectangular areas of 4 km × 4 km according to the systematic sampling interval of 4 km, as shown in Fig. 5. Secondly, in each rectangular sampling area, a suitable local area that could represent the typical forest stand characteristics of the rectangular area was selected to arrange the field plots. This local area was composed of three 400 m × 600 m strips forming a herringbone area. A field plot was arranged in the overlapping area of the three strips, and one field plot was arranged in each of the three strips. According to this method, 4 field plots with the size of 25 m × 25 m were collected in each rectangular area of 4 km × 4 km apart from the restricted access areas NO 3, 4, and 5.

Considering that the research object of this study was coniferous forests, the representative coniferous forests sample plots were selected in the herringbone region of the primary selection. According to the principle of uniform distribution, keeping away from roads and covering different forest ages as much as possible, a total of 76 coniferous forests plots were selected in Wangyedian Forest Farm, including 42 Chinese pine (*Pinus tabuliformis Carr.*) plots, 30 larch (*Larix gmelinii (Rupr.) Kuzen.*) plots and 4 Scots pine (*pinus sylvestre*) plots. The Scots pine plots was classified in non-stratification. The specific plot distribution is shown in the Fig. 5.

During the field work, Real Time Kinematic (RTK) was used to accurately locate the corner and center points of the plot with an error of less than 20 cm. All the trees with DBH greater than 5 cm in the sample plot were measured to record DBH, tree height, crown width and other information. The AGB of the field plots was calculated based on allometric growth equations of tree species. Table 5 presents a summary of the field estimated AGB.

**Table 5:** Summary of the field estimated AGB (Mg/ha)

Category	Number of plots	AGB range	Mean AGB	Standard deviation
Larch	30	29.83–210.16	113.99	45.84
Chinese pine	42	68.59–277.39	153.00	46.89
Scots pine	4	19.52–148.32	84.31	45.55
Non-stratification	76	19.52–277.39	133.99	51.39

#### LiDAR data

The UAV-LiDAR data were acquired from 15 to 30 September 2019 using a RIEGL vux-1 LiDAR sensor mounted on a RC6-2000 UAV. The acquired UAV-LiDAR data covered 19 herringbone sampling areas, which are the herringbone areas selected in the field survey described in "Field data" section. The UAV flight altitude was 280 m above the ground and the flight speed was 4.7 m/s. For laser pulse, only the first return was recorded, and the overall point density was better than 40 points/m^2^. The detailed parameters of UAV-LiDAR are shown in Table 6.

**Table 6:** The main parameters of UAV-LiDAR system

Parameters	Value	Parameters	Value
Wavelength	Near-infrared	Frequency (KHz)	550
Scanning the field of view (°)	330	Flight altitude (m)	280
Scanning angle resolution (°)	0.001	Flight speed (m/s)	4.7
Scanning speed (line/s)	200	Line spacing (m)	200

The preprocessing of LiDAR data included denoising, point cloud classification, elevation normalization and intensity correction. The noise points were removed using the height threshold, then the point clouds after denoising were classified as ground and non-ground based on Digital Elevation Model (DEM) generated by ground point interpolation using irregular triangulation network algorithm. Point cloud normalization was completed based on DEM, which could remove the influence of topographic relief on elevation value of point cloud data. And point clouds with height > 0.2 m were classified as vegetation. Finally, since the point cloud data of UAV-LiDAR have an obvious intensity banding issue, the intensity correction was carried out on the normalized vegetation point cloud to reduce the error.

1 $${I}_{C}={I}_{raw} \frac{{R}_{i}^{2}}{{R}_{ref}^{2}*cos{\theta }_{i}}$$

where ${\mathrm{I}}_{\mathrm{C}}$ is the corrected intensity, ${\mathrm{I}}_{\mathrm{raw}}$ is the raw intensity value, ${\mathrm{R}}_{\mathrm{i}}$ is the range between sensor and target, ${\mathrm{R}}_{\mathrm{ref}}$ is the standard range (e.g. 1000 m) and ${\uptheta }_{\mathrm{i}}$ is the scan angle.

#### Sentinel-2 image

Two cloud-free Sentinel-2B Level-1C images covering the study area on September 17nd, 2019 were downloaded from the Copernicus Scientific Data Hub (CSDB, https://scihub.copernicus.eu/). The Level-1C image is the Top-Of-Atmosphere reflectance product after ortho-rectification and sub-pixel multispectral registration. In this study, Level-2A Bottom-Of-Atmosphere reflectance products were obtained by atmospheric correction using the Sen2cor atmospheric correlation Processor (version 2.8.0). Then, in order to reduce the influence of mountainous terrain, the C model was used for terrain correction (2, 3). All images were resampled to 10 m of spatial resolution.

2 $$L_H = L_T \left( {\frac{\cos (sz) + C}{{\cos (i) + C}}} \right)$$

3 $$\cos i = \cos sz\cos tz + \sin sz\sin tz\cos (sa - ta)$$

C = b/m, where m is slope and b is intercept of the regression equation derived from cos(*i*). *sz* is the solar zenith angle, *sa* is the solar azimuth angle, *tz* is the surface normal zenith angle or the terrain slope, and *ta* is the terrain azimuth angle.

#### Sentinel-1 image

One Sentinel-1B Level-1 Single Look Complex (SLC) image in Interferometric Wide Swath (IW) mode covering the study area was acquired on September 25nd, 2019 from the CSDB. The acquired image has a 5 m range by 20 m azimuth spatial resolution in VV and VH polarizations and is in ascending mode with a mean incident angle of 37.6°.

The preprocessed steps of Sentinel-1 Level-1 SLC image included thermal noise removal, orbital correction, radiometric calibration, deburst, multi-looking, speckle filtering using the refined Lee sigma filter, terrain correction using a range-Doppler terrain correction with the SRTM 1Sec HGT DEM, effective scattering area correction based on local incidence angle, and converting into dB unit. And the image was resampled to 10 m of spatial resolution. In addition, the Sentinel-1 and Sentinel-2 images were co-registered to LiDAR data, and the coordinate system was WGS_1984_UTM_zone_50N.

#### Auxiliary data

1. UAV CCD images
  We obtained UAV CCD orthographic data of the same period and coverage as the airborne LiDAR data (Fig. 5). The spatial resolution of CCD data is 0.2 m. The acquired UAV CCD orthographic images could be used to visually identify larch and Chinese pine, coniferous and broad-leaf forests for the LiDAR sampling plots collection.
2. The subcompartment survey data
  The subcompartment is the basic unit of forest resource planning and design investigation, statistics and management, which contains information such as the area and ownership of various forest lands, forest origin, ecological factors related to forest resources, and natural geographic environmental factors, etc. In this study, the distribution range of coniferous forest tree species was acquired by using the attributes of tree species types from the subcompartment survey data of Wangyedian Experimental Forest farm updated in 2020. Based on this coniferous forest tree species distribution map, LiDAR sampling was carried out.

### Overview of the point-line-polygon framework

**Fig. 6:**
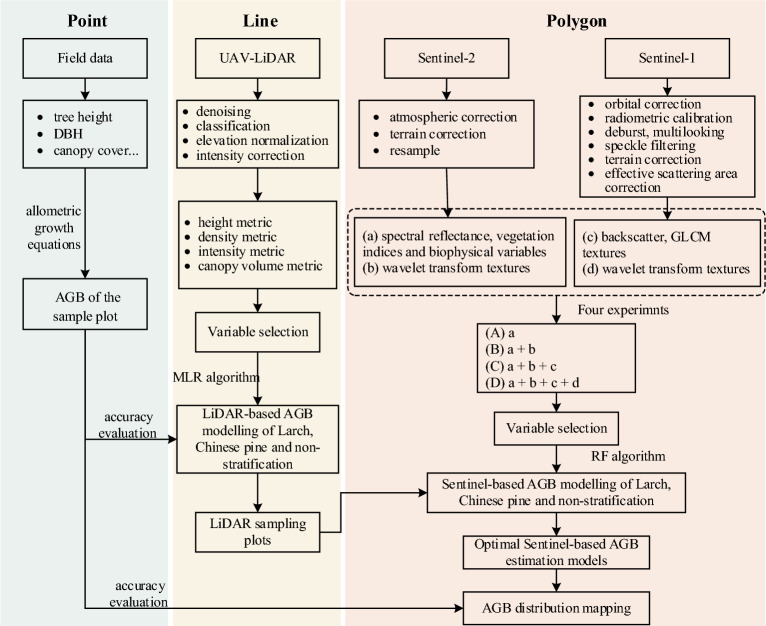
The workflow of this study

We conducted a point-line-polygon framework to support a wall-to-wall high-resolution forest AGB mapping using field plots, UAV-LiDAR strip data and satellite imagery. AGB derived from UAV-LiDAR strip data as a baseline to Sentinel-based AGB models that used LiDAR sampling plots could achieve accuracies. We sought to confirm the feasibility of the LiDAR sampling strategy consistent with the field plots, and whether the use of tree species stratification and WT texture contributed to promote the accuracy of AGB estimation. Figure 6 shows the workflow of this study.

In the point-line-polygon framework, the point denotes ground survey field plot data, the line represents UAV-LiDAR strip data of high-density point clouds, and the polygon represents large-scale full coverage space-borne optical and SAR imagery. The point-line-polygon framework is mainly divided into two stages: point-line part and line-polygon part. We built AGB estimation models of larch, Chinese pine and all coniferous forests (non-stratification) respectively at the two stages. The first stage is to establish the LiDAR-based AGB models using UAV-LiDAR metrics and field plots by multiple linear regression (MLR) method and obtain the AGB_LiDAR_ strip map. Subsequently, the classification-based visual interpretation sampling approach was used to select the LiDAR sampling plots. In the second stage, AGB_LiDAR_ of the selected LiDAR sampling plots is used as the reference AGB data, and the Sentinel-based AGB models are generated by combining Sentinel-1 and Sentinel-2 images by Random Forest (RF) algorithm to produce a wall-to-wall AGB map. Four groups of experiments A, B, C and D are set up to assess the effect of different type features, and two validation sets are used to evaluate the performance of Sentinel-based AGB models.

### Variables derived from LiDAR and Sentinel data

A total of 58 LiDAR metrics (Table 7) including point cloud height, point cloud intensity, point cloud density and canopy volume structure were derived at 1 m resolution from the intensity corrected normalized vegetation cloud points.

**Table 7:** The list of metrics derived from UAV-LiDAR point clouds

Type	Variable	Definition
Height metric (22)	H01, H05, H10, H20, H25, H30, H40, H50, H60, H70, H75, H80, H90, H95, H99	Height percentile, all the normalized point clouds are sorted according to the elevation. HX is the Xth percentile of point cloud height
	Hmax	Maximum height
	Hmin	Minimum height
	Hmean	Mean height
	Hmedian	Median of height
	H.cv	Coefficient of variation of height
	H.ske	Skewness of heights
	H.kur	Kurtosis of heights
Density metric (10)	D01, D02, D03, D04, D05, D06, D07, D08, D09, D10	Canopy return density, the point clouds are divided into ten slices of the same height from low to high. D01 to D10 corresponded to the point density from the lowest slice to the highest
Intensity metric (22)	I01, I05, I10, I20, I25, I30, I40, I50, I60, I70, I75, I80, I90, I95, I99	Intensity percentile, all the normalized point clouds are sorted according to the intensity. IX is the Xth percentile of point cloud intensity
	Imax	Maximum intensity
	Imin	Minimum intensity
	Imean	Mean intensity
	Imedian	Median of intensity
	I.cv	Coefficient of variation of intensity
	I.ske	Skewness of intensities
	I.kur	Kurtosis of intensities
Vegetation Index metric (4)	LAI	Leaf Area Index, $-\frac{\mathrm{cos}\left(\mathrm{ang}\right)\times \mathrm{ln}\left(\mathrm{G}.\mathrm{F}\right)}{\mathrm{k}}$, ang is the average scan angle, G.F is the gap fraction and k is the extinction coefficient
	G.F	Gap Fraction, $\frac{{\mathrm{n}}_{\mathrm{ground}}}{\mathrm{n}}$, ratio of ground points, less than 0.2 m in normalized cloud points, to all normalized cloud points
	C.R.R	Canopy Relief Ratio, $\frac{{\mathrm{H}}_{\mathrm{mean}}-{\mathrm{H}}_{\mathrm{min}}}{{\mathrm{H}}_{\mathrm{max}}-{\mathrm{H}}_{\mathrm{min}}}$
	C.C	Canopy Cover, $\frac{{\mathrm{n}}_{\mathrm{vegfirst}}}{{\mathrm{n}}_{\mathrm{first}}}$, ratio of vegetation cloud points of first echo to all cloud points of first echo

All 197 remote sensing indices were extracted for AGB estimation, with 154 and 43 indicators from optical and SAR, respectively. For Sentinel-2, we extracted 10 spectral bands, 19 vegetation indices, 5 biophysical variables and 120 multi-scale WT textures (Table 8). In terms of Sentinel-1 data, 3 backscatters, 16 spatial domain textures, and 24 multi-scale WT textures (Table 9). The spatial domain texture of VV and VH polarization were calculated using Grey Level Co-occurrence Matrix algorithm [19] with a 3 × 3 window and 45°direction. The multi-scale WT textures, representing frequency domain texture information, were derived by using two-dimensional (2-D) discrete wavelet transform (DWT) algorithm.

**Table 8:** The list of indices derived from Sentinel-2 data

Variable Group	Type (number)	Variable	Definition
a	Spectral reflectance (10)	B2	Blue, 490 nm
		B3	Green, 560 nm
		B4	Red, 665 nm
		B5	Red edge, 705 nm
		B6	Red edge, 749 nm
		B7	Red edge, 783 nm
		B8	Near infrared, 842 nm
		B8a	Near infrared, 865 nm
		B11	Short wave infrared, 1610 nm
		B12	Short wave infrared, 2190 nm
	Vegetation indices (19)	ARVI	Atmospherically resistant vegetation index, $\mathrm{B}8-(2\times \mathrm{B}4-\mathrm{B}2)/\mathrm{B}8+(2\times \mathrm{B}4-\mathrm{B}2)$
		CIg	Chlorophyll Index green, $(\mathrm{B}8/\mathrm{B}3)-1$
		CIre	Chlorophyll Index red edge, $(\mathrm{B}7/\mathrm{B}5) -1$
		II	Infrared index, $(\mathrm{B}8-\mathrm{B}11)/(\mathrm{B}8+\mathrm{B}11)$
		MCARI	Modified chlorophyll absorption ratio index, $\left[\left(\mathrm{B}5-\mathrm{B}4\right)-0.2\times (\mathrm{B}5-\mathrm{B}3)\right]\times (\mathrm{B}5-\mathrm{B}4)$
		S2REP	Sentinel-2 red-edge position index, $705+35\times \left[\frac{\left(\mathrm{B}4+\mathrm{B}7\right)}{2}-\mathrm{B}5\right]\times (\mathrm{B}6-\mathrm{B}5)$
		MidIR	Infrared index, $\mathrm{B}11/\mathrm{B}12$
		MSI	Moisture Stress Index, $\mathrm{B}11/\mathrm{B}8$
		NDVI	Normalized difference vegetation index, $(\mathrm{B}8-\mathrm{B}4) / (\mathrm{B}8 +\mathrm{ B}4)$
		NDI45	Normalized difference vegetation index wi band4 and band5, $(\mathrm{B}5-\mathrm{B}4) / (\mathrm{B}5 +\mathrm{ B}4)$
		RVI	Ratio vegetation index, $(\mathrm{B}8 /\mathrm{ B}4)$
		SAVI	Soil adjusted vegetation index, $1.5\times (\mathrm{B}8 -\mathrm{ B}4) / 8\times (\mathrm{B}8 +\mathrm{ B}4 + 0.5)$
		IPVI	Infrared percentage vegetation index, $\mathrm{B}8 / (\mathrm{B}8 +\mathrm{ B}4)$
		PVI	Perpendicular vegetation index, $\mathrm{sin}(45^\circ )\times \mathrm{B}8 -\mathrm{ cos}(45^\circ )\times \mathrm{B}4$
		PSSRa	Pigment specific simple ratio chlorophyll index, $\mathrm{B}7/\mathrm{B}4$
		PSRI	Plant Senescence Reflectance Index, $(\mathrm{B}4-\mathrm{B}3)/\mathrm{B}6$
		REIP	Red-edge infection point index, $700+40\times \left[\frac{\left(\mathrm{B}4+\mathrm{B}7\right)}{2}-\mathrm{B}5\right]/(\mathrm{B}6-\mathrm{B}5)$
		TNDVI	Transformed Normalized Difference Vegetation Index, $\sqrt{\frac{\mathrm{B}8-\mathrm{B}4}{\mathrm{B}8+\mathrm{B}4}+0.5}$
		VARI	Visible light atmospheric impedance vegetation index, $(\mathrm{B}3-\mathrm{B}4)/(\mathrm{B}3+\mathrm{B}4-\mathrm{B}2)$
	Biophysical variables (5)	LAI	Leaf area index
		FVC	Fraction of vegetation cover
		FAPAR	Fraction of absorbed photo synthetically active radiation
		Cab	Chlorophyll content in the leaf
		Cwc	Canopy water content
b	Multi-scale WT texture of Sentinel-2 (120)	a_i_Bj	Approximate texture
		h_i_Bj	Horizontal texture
		v_i_Bj	Vertical texture
		d_i_Bj	Diagonal texture

Note: i represents the level of wavelet decomposition, from 1 to 3. Bj represents the band of Sentinel-2, from B2 to B8, B8a and B11, B12

**Table 9:** The list of variables derived from Sentinel-1 data

Variable group	Type (number)	Variable	Definition
c	Backscatter (3)	VV	Backscatter coefficient of VV
		VH	Backscatter coefficient of VH
		VH/VV	Ratio of VH to VV
	Spatial texture (16)	VH_mean, VV_mean	Mean
		VH_ent, VV_ent	Entropy
		VH_con, VV_con	Contrast
		VH_dis, VV_dis	Dissimilarity
		VH_var, VV_var	Variance
		VH_cor, VV_cor	Correlation
		VH_hom, VV_hom	Homogeneity
		VH_asm, VV_asm	Angular second moment
d	Multi-scale WT texture of Sentinel-1 (24)	a_m_Bn_sar	Approximate texture
		h_m_Bn_sar	Horizontal texture
		v_m_Bn_sar	Vertical texture
		d_m_Bn_sar	Diagonal texture

Note: m represents the level of wavelet decomposition, from 1 to 3. n = 1 or 2, B1 represents the VH channel, B2 represents the VV channel

WT can decompose signals at different scales. DWT is the discretization of scale factor a and translation factor b in continuous wavelet transform (CWT). The formula of CWT $W_{f(a,\tau )}$ is as follows.

4 $$W_{f(a,\tau )} = \int_{ - \infty }^\infty {f(t)\psi_{a,\tau } } (t)dt = \int_{ - \infty }^\infty {f(t)\frac{1}{{\sqrt {a} }}} \psi (\frac{t - \tau }{a})dt$$

where α is the scale factor, τ is the translation factor, $\psi_{a,\tau } (t)$ is the wavelet basis function, and $W_{f(a,\tau )}$ represents the CWT.

Generally, the low and high frequencies of the signal decomposition are transformed to the discrete wavelet basis by transforming the α and τ into a power series structure.

5 $$a = a_0^j$$

6 $$\tau = ka_0^j \tau_0$$

7 $$\psi_{j,k} (t) = a_0^{ - \frac{j}{2}} \psi (a_0^{ - j} t - k\tau_0 )$$

where $a_0 \ne {1}$, $\tau_{0}$ is a constant and $\psi_{j,k} (t)$ is the corresponding discrete wavelet basis of j, k ∈ Z.

Then, the formula of final DWT is as follows.

8 $$W_{f(j,k)} = \int {f(t)\overline{{\psi_{j,k} (t)}}d} (t)$$

**Fig. 7:**
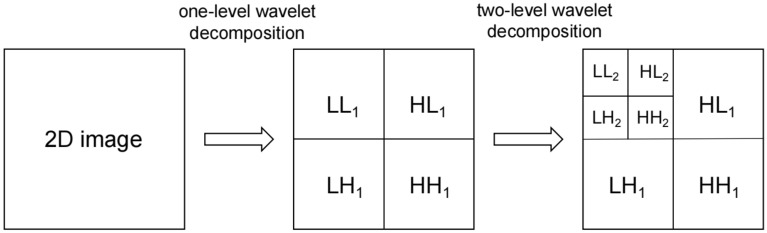
Schematic diagram of 2-D DWT

2-D DWT algorithm is to transform 2-D images into row and column data respectively, and then carry out one-dimensional discrete wavelet transform. The principle of 2-D DWT is shown in Fig. 7. After the one-level wavelet decomposition, the approximate features (LL_1_) of the low-frequency subband and the detailed features of the horizontal (HL_1_), vertical (LH_1_) and diagonal directions (HH_1_) of the high-frequency subband are extracted. In the second level wavelet decomposition, the approximate low-frequency components of the one-level wavelet decomposition are decomposed by the same operation again, and the approximate low-frequency component (LL_2_) and three detailed high-frequency components (LH_2_, HL_2_ and HH_2_) of the second level wavelet decomposition are further obtained. And so on.

In this study, we selected the Sym5 algorithm as the wavelet basis function to carry out the three-level wavelet decomposition, then extracted the textures of 10 spectral bands and dual polarization at the corresponding scale.

### Variable selection

The RF algorithm is insensitive to multiple collinearity, and the results are robust with respect to missing data and non-equilibrium data [24]. It is actually an improved bagging approach that uses a CART tree as a model in Bagging. In addition to classification and regression, the RF algorithm can determinate the relative importance of different variables and serve as a high-dimensional feature selection tool, which has two evaluation indexes, %Inc MSE and Inc Node Purity. The higher the values of %Inc MSE and Inc Node Purity are, the greater the importance is.

In the two stages of this point-line-polygon framework, we all used the Pearson correlation analysis and RF algorithm to determine the best combinations of the variables. First, variables that were significantly (p < 0.05) correlated with reference AGB were selected. Afterwards, the importance of the obtained preliminary candidate variables was ranked based on RF algorithm, and the first n variables whose importance differs greatly from the subsequent variables were selected according to the rank. The RF algorithm implemented in R language was used to model and evaluate the importance of the variables. For importance ranking, ntree was set to 1000 and mtry was set to one third of the number of variables involved.

### Forest AGB modelling from LiDAR data

In the point-line part, the relationship between field-based AGB and LiDAR metrics was explored using MLR model. 42 Chinese pine plots, 30 larch plots and all 76 coniferous forests plots were used to build and validate the LiDAR-based AGB models based on the optimal LiDAR metric combinations after variable selection under Chinese pine, larch and non-stratification scenarios.

Considering the limited number of field plots, K-fold cross validation method was used to evaluate the model. K-fold cross validation means that the dataset is randomly divided into K groups, among which the training samples are K − 1 folds and the validation dataset is one fold, and this process is iterated for k times. In this study, we used the leave-one-out cross-validation, that is K = n, for calculating root mean squared error (RMSE), relative RMSE (rRMSE), mean absolute error (MAE) and correlation coefficient (r), to assess the performance of the models.

9 $$r=\sqrt{{R}^{2}}=\sqrt{1-\frac{{\sum }_{i=1}^{n} {\left(\mathrm{y}-\overline{y}\right)}^{2}}{{\sum }_{i=1}^{n} {\left(\widehat{y}-\overline{y}\right)}^{2}}}$$

10 $$RMSE =\sqrt{\frac{\sum_{i=1}^{N}{\left(\widehat{y}-y\right)}^{2}}{\mathrm{N}}}$$

11 $$rRMSE = \frac{RMSE}{\overline{\mathrm{y}} }$$

12 $$MAE=\frac{1}{N}\sum_{i=1}^{N}\left|\widehat{y}-y\right|$$

where $y$ represents the measured value, $\widehat{y}$ represents the predicted value, $\overline{\mathrm{y} }$ represents the measured average value of $\widehat{y}$, n represents the number of plots.

### LiDAR sampling plots acquisition

In the point-line-polygon framework, obtaining the LiDAR sampling plots is the key step, which affects the AGB estimation accuracy. In order to reduce the error in the up-scaling process, we completed the LiDAR plot sampling according to the criteria consistent with the field plot sampling. The stratified sampling scheme was adopted in the study. The larch, Chinese pine and other coniferous forests were identified by visual interpretation mainly based on a 0.2 m UAV CCD orthographic images, assisted by the subcompartment data. During the sampling process, the sampling plots were selected in accordance with the principles of uniform distribution and away from road within the coverage area of the UAV-LiDAR strip products. Then, with the selected LiDAR sampling point as the center, a rectangular sampling plot of 25 m * 25 m was generated, and the direction of the sampling plot was due south due north. These LiDAR sampling plots were used as training data for modeling at regional scale in the second stage.

### Biomass up-scaling and assessment

In the line-polygon part, the AGB_LiDAR_ of LiDAR sampling plots was used as the reference AGB, and combined with the variables extracted from Sentinel-1 and Sentinel-2 images, the Sentinel-based AGB models of larch, Chinese pine and non-stratification were established respectively using RF methods after variable selection.

Four experiments were conducted to analyze the suitability of different combinations of variable groups in AGB mapping: (A) variable group a; (B) variable group a and b; (C) variable group a, b and c; (D) variable group a, b, c and d.

**Fig. 8:**
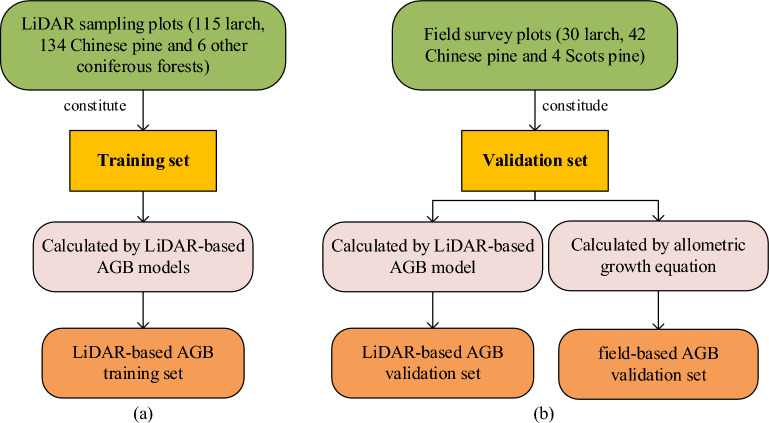
Establishment of training set (**a**) and validation set (**b**)

The LiDAR sampling plots were used as training samples to build Sentinel-based AGB models of larch, Chinese pine and non-stratification (Fig. 8). The field survey plots, including 30 larch, 42 Chinese pine and 4 Scots pine plots, were used as validation samples to evaluate the performance of these Sentinel-based AGB models. In order to fully evaluate the applicability and accuracy of estimated AGB based on the point-line-polygon framework, two validation sets were established using the same validation samples. The AGB value of first validation set (LiDAR-based AGB validation set) was acquired from the LiDAR-based models, and the second one (field-based AGB validation set) was calculated by allometric growth equation (Fig. 8).

The LiDAR-based AGB validation set is used to verify the accuracy of estimated coniferous forests AGB under the point-line-polygon framework. The field-based AGB validation set, belonging to the point scale, can be used to analyze error transmission and accumulation during the up-scaling process. For two validation sets, we all used r, RMSE, rRMSE, and MAE to assess the performance of the Sentinel-based models in four scenarios.

## Data Availability

The datasets generated and/or analysed during the current study are not publicly available due [the funded project is under development] but are available from the corresponding author on reasonable request.

## Abbreviations

UAV: Unmanned aerial vehicle
AGB: Aboveground biomass
WT: Wavelet transform
LiDAR: Light Detection and Ranging
RTK: Real Time Kinematic
DEM: Digital Elevation Model
SLC: Single Look Complex
MLR: Multiple linear regression
RF: Random Forest
2-D: Two-dimensional
DWT: Discrete wavelet transform
RMSE: Root mean squared error
rRMSE: Relative RMSE
MAE: Mean absolute error
r: Correlation coefficient
